# Solution NMR Structure of Hypothetical Protein CV_2116 Encoded by a Viral Prophage Element in *Chromobacterium violaceum*

**DOI:** 10.3390/ijms13067354

**Published:** 2012-06-14

**Authors:** Yunhuang Yang, Theresa A. Ramelot, John R. Cort, Maite Garcia, Adelinda Yee, Cheryl H. Arrowsmith, Michael A. Kennedy

**Affiliations:** 1 Department of Chemistry and Biochemistry and the Northeast Structural Genomics Consortium, Miami University, Oxford, OH 45056, USA; E-Mails: yangy9@muohio.edu (Y.Y.); ramelota@muohio.edu (T.A.R.); 2 Biological Sciences Division, Pacific Northwest National Laboratory, Richland, WA 99352, USA; E-Mail: john.cort@pnl.gov; 3 Campbell Family Cancer Research Institute, Ontario Cancer Institute and the Northeast Structural Genomics Consortium, University Health Network, Toronto, ON, M5G 2C4, Canada; E-Mails: mgarcia@uhnres.utoronto.ca (M.G.); aayee@uhnres.utoronto.ca (A.Y.); carrow@uhnres.utoronto.ca (C.H.A.)

**Keywords:** CV_2116, *Chromobacterium violaceum*, NMR, solution structure, structural genomics, bacteriophage tail assembly, prophage, lateral gene transfer

## Abstract

CV_2116 is a small hypothetical protein of 82 amino acids from the Gram-negative coccobacillus *Chromobacterium violaceum*. A PSI-BLAST search using the CV_2116 sequence as a query identified only one hit (E = 2e^−07^) corresponding to a hypothetical protein OR16_04617 from *Cupriavidus basilensis* OR16, which failed to provide insight into the function of CV_2116. The *CV_2116* gene was cloned into the p15TvLic expression plasmid, transformed into *E. coli*, and ^13^C- and ^15^N-labeled NMR samples of CV_2116 were overexpressed in *E. coli* and purified for structure determination using NMR spectroscopy. The resulting high-quality solution NMR structure of CV_2116 revealed a novel α + β fold containing two anti-parallel β-sheets in the *N*-terminal two-thirds of the protein and one α-helix in the *C*-terminal third of the protein. CV_2116 does not belong to any known protein sequence family and a Dali search indicated that no similar structures exist in the protein data bank. Although no function of CV_2116 could be derived from either sequence or structural similarity searches, the neighboring genes of *CV_2116* encode various proteins annotated as similar to bacteriophage tail assembly proteins. Interestingly, *C. violaceum* exhibits an extensive network of bacteriophage tail-like structures that likely result from lateral gene transfer by incorporation of viral DNA into its genome (prophages) due to bacteriophage infection. Indeed, *C. violaceum* has been shown to contain four prophage elements and *CV_2116* resides in the fourth of these elements. Analysis of the putative operon in which CV_2116 resides indicates that CV_2116 might be a component of the bacteriophage tail-like assembly that occurs in *C. violaceum*.

## 1. Introduction

*Chromobacterium violaceum* is a Gram-negative non-sporulating coccobacillus. It produces a natural antibiotic called violacein, which may be useful for treatment of colon and other cancers [1]. The complete genome of *C. violaceum*, which was published in 2003 [2], contains four identified integrated viral genomes (prophages) [3]. Among these prophages, CvP4 is composed of 51 ORFs spanning *CV2114* to *CV2150*, including *CV_2116*, which encodes a hypothetical protein of 82 residues (UniProtKB/TrEMBL ID Q7NW74_CHRVO). To date, CV_2116 does not belong to any known protein families (Pfam 26.0, November, 2011) and is simply annotated as a putative uncharacterized protein [4]. This hypothetical protein was selected for structure determination as a target by the Northeast Structural Genomics (NESG) consortium (NESG Target ID CvT4). A goal of the United States National Institutes of Health’s Protein Structure Initiative (PSI) during the 10-year period spanning the PSI-1 and PSI-2 phases was to establish an understanding of all protein sequence/protein fold relationships that occur in nature with an emphasis on determining at least one experimental protein structure for every identified protein sequence family. However, not all proteins predicted by genome sequencing efforts belong to existing sequence families. Such proteins are referred to as “singletons”, which is currently the case for CV_2116. Therefore, despite not belonging to an existing protein family, knowledge of the three-dimensional structure of the hypothetical CV_2116 protein helps to fill in a missing part of the sequence-protein fold space that occurs in nature.

## 2. Results and Discussion

**Figure 1:**
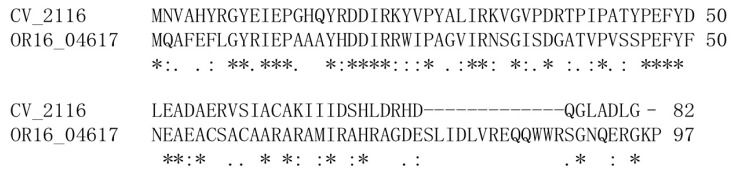
Sequence alignment of CV_2116 to OR16_04617 generated using ClustalW2 [7].

A sequence similarity search against CV_2116 using PSI-BLAST identified only one hit, a hypothetical protein OR16_04617 from *Cupriavidus basilensis* OR16 (E-value of 2e^−07^) with sequence identity of 43% over all 82 residues. Likewise, a PSI-BLAST search using OR16_04617 as the query identified CV_2116 as the only significant sequence match. Figure 1 shows the sequence alignment of CV_2116 to OR16_04617. The other two proteins identified by PSI-BLAST had E-values greater than 1.0, including GFO_0138 (E-value of 1.3) from *Gramella forsetti* (UniProtKB/TrEMBL ID A0LXN2_GRAFK) [5] with sequence identity of 33% over 60 residues, and Ribonuclease HII (E-value of 6.3) from *Bacillus coagulans* (UniProtKB/TrEMBL ID G2TJM8_BACCO) [6] with sequence identity of 36% over 42 resides. The low sequence similarity to other proteins indicated that CV_2116 is not a highly conserved protein domain, and the single PSI-BLAST hit to OR16_04617 failed to provide any insight into the function of CV_2116.

**Figure 2:**
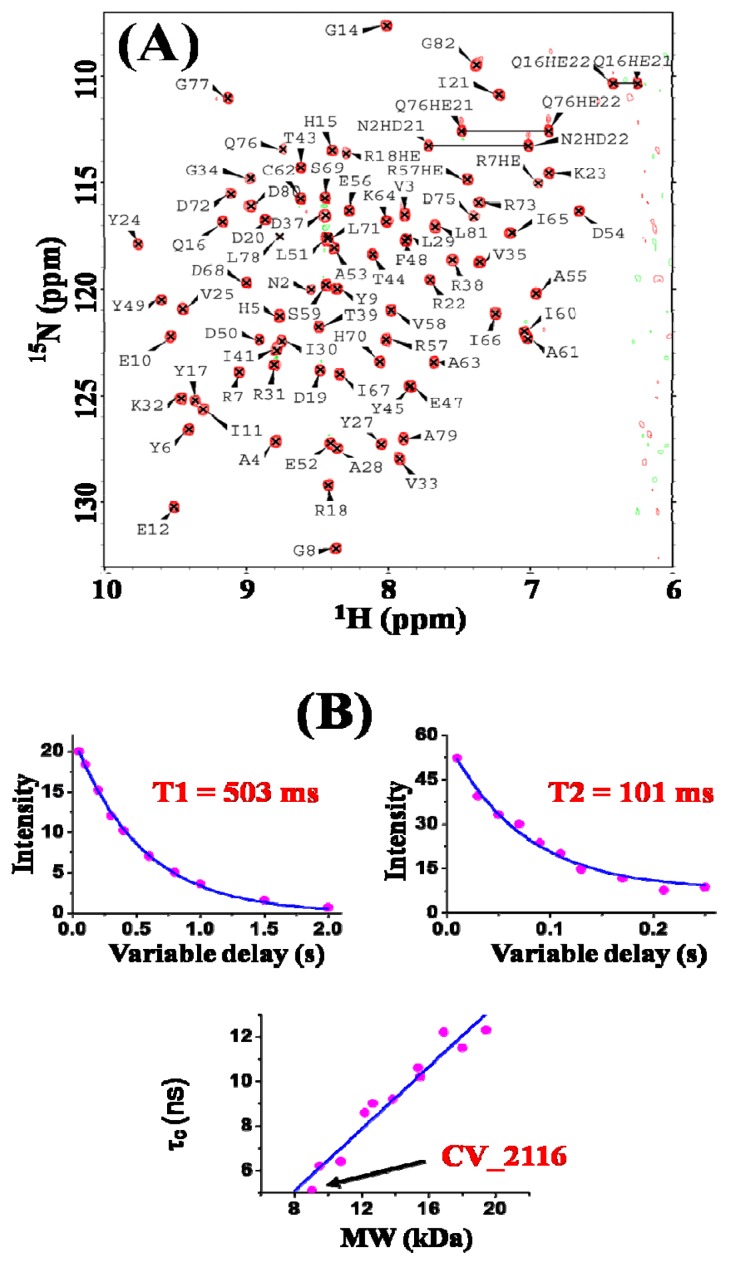
(**A**) Two dimensional ^1^H-^15^N HSQC spectrum of about 0.8 mM CV_2116 in 10 mM Tris (pH 7.0), 300 mM NaCl, 10 mM DTT, 1 mM benzamidine, 0.01% NaN_3_, 1× inhibitor cocktail (Roche Applied Science), 95% H_2_O/5% D_2_O collected at 20 °C on a Varian Inova 600 MHz NMR spectrometer. Backbone resonance assignments are labeled with one-letter amino acid codes followed by their sequence numbers. Assigned side chain NH^□^ resonances of Arg (aliased), and side chain NH_2_ resonances of Asn and Gln are also indicated; (**B**) 1D ^15^N *T*_1_ and *T*_2_ relaxation data for CV_2116. (Top): ^15^N *T*_1_ and *T*_2_ values were extracted by plotting the decay of integrated ^1^H^N^ intensity between 8.5 and 10.5 ppm and fitting the curves with standard exponential equations. (Bottom): Plot of rotational correlation time, τ_c_ (ns), versus protein molecular weight (kDa) for known monomeric NESG targets of ranging size (taking into account isotope enrichment as well as affinity tags in the sequence). ^15^N *T*_1_/*T*_2_ data for all monomeric proteins used for the τ_c_ *vs*. MW plot were obtained on the same Varian Inova 600 MHz spectrometer at 298 K.

A high quality solution structure of CV_2116 was determined by NMR spectroscopy. CV_2116 exhibited an excellent ^1^H-^15^N-HSQC spectrum (Figure 2A) indicative of a well-behaved and structured protein in solution. The rotational correlation time of CV_2116 was determined from average ^15^N relaxation times extracted from 1D ^15^N-edited *T*_1_ and *T*_2_ experiments of the NC sample recorded on a 600 MHz spectrometer at 298 K. The overall isotropic rotational correlation time (τ_c_) of 6.2 ± 0.5 ns derived from *T*_1_ and *T*_2_ measurements confirmed its monomeric state in solution based on a curve of molecular weight versus τ_c_ from other proteins characterized in our laboratory with molecular weights under 20 kDa as shown in Figure 2B [8].

**Figure 3:**
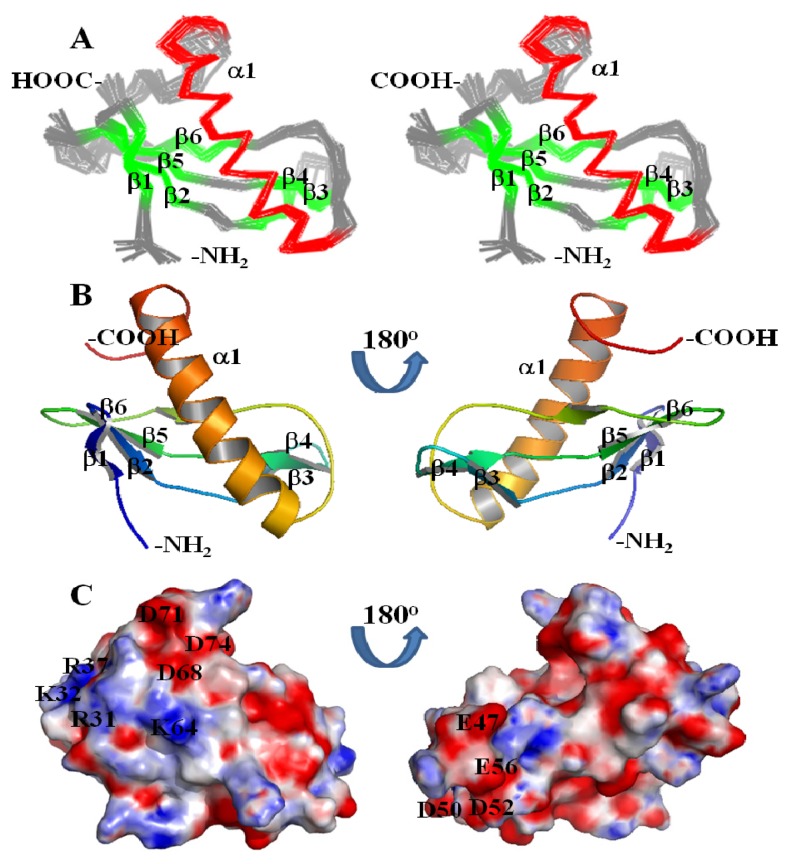
(**A**) Stereoview of the superimpositions of the 20 lowest energy structures of CV_2116. The six β-strands are colored green, α-helix colored red, and the *N*-terminal tail, *C*-terminal tail and loop regions are colored grey; (**B**) Rainbow-colored ribbon representation of the lowest energy CV_2116 structure from the ensemble shown in (**A**). All secondary structural elements are labeled; (**C**) Electrostatic potential surface diagram for identical orientation as in (**B**). A positively charged region (R31, K32, R37 and K64) and a negatively charged region (D68, D71 and D74) are denoted in the left panel, while another negatively charged region (E47, D50, D52 and E56) is denoted in the right panel.

Figure 3A shows the stereoview of the superimposition of the 20 lowest energy structures of CV_2116. The secondary structural elements are locally and globally well defined. CV_2116 contains two anti-parallel β sheets in the *N*-terminal two-thirds of the protein and one α helix in the *C*-terminal third of the protein (Figure 3B). The first β sheet is composed of four β-strands (β1, A4-Y6; β2, Y9-E12; β5, L29-K32; β6, I41-T44) in the order of β1-β2-β5-β6. The short β6 β-strand is present, as predicted by PROCHECK 3.5.4 [9,10], in 13 out of 20 structures in the final NMR ensemble.

Two β-strands (β3, H15-R18 and β4, K23-P26) compose the second anti-parallel β sheet. The 21-residue helix (E52-D72) in the *C*-terminal third of the protein flanks the two anti-parallel β sheets. Analysis of the NOEs and tertiary structure of CV_2116 revealed that Y6 from β1, Y9 from β2, I30 from β5, T44 from β6, and A63, I66, and I67 from α-helix constitute the hydrophobic core contacts to the first β sheet, whereas few NOEs between Y24 from β4 and A55 from the α-helix were observed to the second β sheet. Two longer loops, one between β5 and β6 (V33-P40) and one between β6 and α-helix (E52-D72), and the *C*-terminal tail (R73-G82) were flexible and disordered in the NMR structure. Structural statistics for CV_2116 are presented in Table 1.

CV_2116 adopted an apparently novel fold without any current matches in the CATH [11] and SCOP [12] structural classification databases. Comparison of the three-dimensional structure of CV_2116 using Dali (v.3) [13] revealed little similarity to any known protein structures in the PDB. Three proteins, peptide chain release factor (PDB ID: 3D5C-X), UDP-*N*-acetylmuramate-alanine ligase (PDB ID: 1P31-A), and hyponastic leave 1 (PDB ID: 2L2N-A), had the highest *Z* scores of 3.7, despite the fact that they had low sequence identities of 8%, 8% and 5%, respectively. No biochemical or biological functions could be derived from the structural comparisons with the known structures in the PDB.

The electrostatic surface diagram of CV_2116 is shown in Figure 3C. Positive and negative regions were observed on one side of the protein while one negative region was present on the opposite side. So far, no potential functions of these regions have been identified.

Although the function of the hypothetical CV_2116 protein could not be predicted by either sequence or structure similarity searches, the *CV_2116* gene from *C. violaceum* is sandwiched between several neighboring genes that encode for apparent bacteriophage tail-like assembly proteins. In fact, *C. violaceum* has been observed to exhibit an unusual and extensive organization of bacteriophage tail-like particles [14]. The locus two genes before *CV_2116*, namely *CV_2114,* encodes a probable pyocin R2_PP tail fiber protein. R-type pyocins are a component of the non-flexible tail structure of bacteriophage [15] The gene immediately preceding *CV_2116*, namely *CV_2115*, encodes a protein homologous to other bacteriophage J tail proteins belonging to the protein family PF04865, which are involved in baseplate assembly. Among the genes immediately following *CV_2116*, *CV_2117* encodes a protein homologous to other bacteriophage V proteins (PF04717), which is also involved in baseplate assembly [16] and *CV_2118* is homologous to several members of PF05954, which are phage late control gene D proteins. Hence, it is reasonable to speculate that CV_2116 is a component of a bacteriophage-like tail assembly specific to *C. violaceum*.

Previous analysis of the *C. violaceum* genome has shown that it contains four prophages denoted CvP1 through CvP4 [3]. The latter, CvP4, is composed of 51 ORFs (*CV_2114 to CV_2150*) and is thought to be a chimeric prophage containing ORFs with similarity to different phages. Specifically, it has been suggested that the tail and tail fiber genes, which include *CV_2114-CV_2118*, are similar to P2-like phage genes [14], and in particular, that these genes exhibit similarity to those found in a P2-like prophage of *Salmonella enterica* subsp. *enterica* serovar Typhi, which are the so-called CT18 phages [14,17,18]. Collectively, it has been speculated that CvP4 was responsible for introducing a number of genes from *S. enterica* into the *C. violaceum* genome. A PSI-BLAST search of CV_2117 indicated ~100 hits to proteins with >30% identity, including NP_456044 from *S. enterica* subsp. *enterica* serovar Typhi str. CT18, which had 41% identity with 96% coverage and E = 9e^−40^. Examination of the genes surrounding NP_456044 indicated a putative operon containing 10 consecutive genes spanning NP_456044 to NP_456053, all encoding proteins involved in bacteriophage tail-like assembly. Examination of the strongest hit PSI-BLAST hit (E = 1e^−105^, 72% sequence identity) against CV_2117, namely YP_003745638 from *Ralstonia solanacearum* CFBP2957, which is an aerobic non-sporing, Gram-negative plant pathogenic bacterium, also revealed a putative operon with a collection of genes spanning YP_003745640 to YP_003745645 that are all annotated as bacteriophage tail-like assembly proteins. These comparisons further support the speculation that CV_2116 is likely a novel gene of prophage origin that plays a structural or functional role in bacteriophage tail-like assembly in *C. violaceum.*

## 3. Experimental Section

### 3.1. Preparation of Protein Samples

The full-length gene for CV_2116 from *C. violaceum* was cloned into the expression plasmid p15TvLic including an additional *N*-terminal hexa-histidine tag and TEV cleavage site (22 residues, MGSSHHHHHHSSGRENLYFQGH), and the plasmid was transformed into *E. coli* BL21 (DE3)-RIPL (Stratagene). *E. coli* cells were grown in 0.5 L of 2 × M9 minimal medium containing ^15^NH_4_Cl and ^13^C-glucose as the sole nitrogen and carbon sources, and supplemented with ZnSO_4_, thiamine, and biotin. Cells were grown at 37 °C to an OD_600_ of 1.0, followed by addition of 1 mM isopropyl β-d-thiogalactoside. The temperature was reduced to 15 °C, and cells grew overnight before harvesting. Cell pellets were lysed by sonication in a buffer containing 500 mM NaCl, 20 mM Tris and 5 mM imidazole (pH 8.0). The proteins were isolated from the lysate by nickel affinity chromatography (Qiagen) after washing the beads three times with five column volumes of 500 mM NaCl, 20 mM Tris (pH 8.0), 30 mM imidazole. Protein was eluted with five column volumes of 500 mM imidazole added to the washing buffer. For NMR structure determination, the hexahistidine tag of CV_2116 was cleaved with TEV protease and the mixture passed through a nickel affinity column. The purified ^13^C/^15^N labeled protein (referred to as the NC sample) was concentrated, and buffer was exchanged by ultrafiltration and dilution/reconcentration into the NMR buffer containing 10 mM Tris (pH 7.0), 300 mM NaCl, 10 mM DTT, 1 mM benzamidine, 0.01% NaN_3_, 1× inhibitor cocktail (Roche Applied Science), 95% H_2_O/5% D_2_O. Following the same procedure, a uniformly 100% ^15^N-labeled and 7% biosynthetically directed ^13^C (NC7) CV_2116 sample was also prepared for stereospecific assignments of isopropyl methyl groups of Val and Leu residues.

### 3.2. NMR Spectroscopy and Data Collection

NMR spectra were acquired on 250 μL NC and NC7 samples in 5 mm Shigemi NMR tubes at 293 K on Varian Inova 600 and 750 MHz NMR spectrometers. Backbone and sidechain resonance assignments were obtained from the analysis of ^15^N-HSQC (Figure 3A), ^13^C-HSQC, HNCO, HNCA, HN(CO)CA, HNCACB, CBCA(CO)NH, C(CO)NH, HC(CO)NH, HBHA(CO)NH, HCCH-COSY, HCCH-TOCSY and (H)CCH-TOCSY spectra. Stereospecific assignments of isopropyl methyl groups of Val and Leu residues were determined from characteristic ^1^H-^13^C coupling patterns in a high resolution ^1^H-^13^C HSQC of the NC7 sample [19]. NOE distance restraints were derived from a ^15^N-edited NOESY-HSQC (mixing time τ_m_ = 70 ms) and two ^13^C-edited NOESY-HSQC (τ_m_ = 70 ms) optimized for either aliphatic or aromatic carbon detection. Additional NOEs were assigned from a 4D ^13^C-^13^C-HMQC-NOESY-HMQC (τ_m_ = 70 ms) recorded after lyophilization and dissolution into pure D_2_O. Spectra were processed by NMRPipe and analyzed with Sparky 3.110 [20]. Nearly complete resonance assignments (99.3%) were determined. NMR assignments, raw NOESY FIDs, and NOESY peak lists have been deposited in the BioMagResBank (BMRB accession number 16,521).

### 3.3. NMR Structure Calculation

The solution NMR structure of CV_2116 was initially calculated using both AutoStructure [21] and CYANA 2.1 [22]. Inputs for calculation were resonance assignments, NOESY peak lists from the four NOESY spectra that included peak intensities, and TALOS-derived dihedral restraints for ϕ and ψ dihedral angles. NOE assignments were initially made automatically in AutoStructure and CYANA and the assignments refined manually. NMR RPF quality assessment scores were used to assess “goodness of fit” between calculated structures and NOESY peaks lists and were used to guide manual and iterative refinement of NOESY peak picking. The NOE-derived distance constraints, dihedral constraints, and hydrogen-bond constraints derived from AutoStructure were converted to Xplor/CNS format using PDBStat and the upper bounds for the NOEs were increased by 10%. These constraints were used to calculate 150 structures using Xplor-NIH (version 2.25) with a standard simulated annealing protocol followed by refinement of the 20 lowest energy structure using the Xplor-NIH protocol [23,24]. For the final NMR ensemble, the 20 lowest energy structures were deposited in the Protein Data Bank (PDB ID, 2KON). NMR structure quality analysis and structural statistics were performed using the PSVS [25] and RPF [26] software packages. Structural statistics for CV_2116 are presented in Table 1.

## 4. Conclusions

In conclusion, protein sequence analysis indicated that the hypothetical protein CV_2116 from *C. violaceum* is not highly conserved among known protein families. The three-dimensional structure of CV_2116 was determined using solution state NMR spectroscopy and analysis of the structure indicated that CV_2116 adopted a novel fold containing two anti-parallel β-sheets and one α-helix. Despite determination of a high quality three-dimensional structure, no biochemical or biological function of CV_2116 could be predicted based on sequence similarity or structural alignment searches. However, analysis of the genes neighboring *CV_2116* indicated that it resides in a putative operon that encodes for five proteins that are likely involved in bacteriophage tail-like assembly, and that the gene and its associated operon is of prophage origin, thereby giving *C. violaceum* distinctly bacteriophage-like characteristics that include the occurrence of a bacteriophage tail-like assembly. In closing, the analysis of CV_2116 from *C. violaceum*, which is both a hypothetical protein and a singleton, is typical of the challenge that is often encountered when researchers attempt to construct a hypothesis regarding the biochemical or cellular function of a hypothetical and/or singleton protein exclusively from sequence and structural information when little other knowledge about the protein is available.

## Figures and Tables

**Table 1:** Summary of NMR and structure statistics for CV_2116 protein from *Chromobacterium violaceum* a.

**Conformationally-Restricting Constraints** b	
**Distance Constraints**	

Intra-residue (I = j)	306
Sequential (|i–j| = 1)	357
Medium-range (1 < |i–j| < 5)	193
Long-range (|i–j| ≥ 5)	419
Total	1275
Distance constraints per residue	15.5
Hydrogen bond constraints	
Long-range (|i–j| ≥ 5)/total	20/56
Dihedral angle constraints	92
Total number of restricting constraints	1423
Number of constraints per residue	5.4

**Residue Constraint Violations** b	
**Average Number of Distance Violations Per Structure**	

0.1–0.2 Å	4.85
0.2–0.5 Å	1.15
>0.5 Å	0
Average RMS distance violation/constraint (Å)	0.01
Maximum distance violation (Å)	0.29

**Average Number of Dihedral Angle Violations Per Structure**	

1–10°	0.7
>10°	0
Average RMS dihedral angle violation/constraint (degree)	0.15
Maximum dihedral angle violation (degree)	2.60

**RMSD from Average Coordinates (Å)** c	

Backbone/Heavy atoms	0.5/0.9

**Ramachandran Plot Statistics** c	

Most favored regions (%)	96.3
Additional allowed regions (%)	3.6
Generously allowed (%)	0.1
Disallowed regions (%)	0

**Global Quality Scores (raw/*Z*-score)** b	

Verify3D	0.24/−3.53
Prosall	0.30/−1.45
Procheck(phi-psi) c	0.21/1.14
Procheck(all) c	0.23/1.36
Molprobity clash	16.49/−1.30

**RPF Scores** d	

Recall	0.98
Precision	0.91
F-measure	0.94
DP-score	0.80

a Structure statistics were computed for the ensemble of 20 deposited structures;

b Calculated using PSVS 1.4 program. Average distance violations were calculated using the summation of r^−6^. Residues 1–82 were analyzed;

c Ordered residues ranges (with sum of phi and psi > 1.8): 3–5, 8–13, 16–32, 40–45, 51–71, 79–81;

d PF scores reflected the goodness-of-fit of the final ensemble of structures including disordered residues to the NMR data.
